# Ceramics and Nanostructures for Energy Harvesting and Storage

**DOI:** 10.3390/nano13222912

**Published:** 2023-11-08

**Authors:** Alexander Tkach, Olena Okhay

**Affiliations:** 1 CICECO-Aveiro Institute of Materials, Department of Materials and Ceramic Engineering, University of Aveiro, 3810-193 Aveiro, Portugal; 2 TEMA-Centre for Mechanical Technology and Automation, Department of Mechanical Engineering, University of Aveiro, 3810-193 Aveiro, Portugal; olena@ua.pt; 3 LASI-Intelligent Systems Associate Laboratory, 4800-058 Guimaraes, Portugal

In recent years, the worldwide research in the field of energy harvesting and storage has focused on the development of clean and sustainable methods that can respond to the rising energy demands of humankind. To enable the transformation from a fossil fuel-based to a low-carbon-based socio-economical epoch, the development of new materials with refined characteristics is necessary. These characteristics include, for example, an enhancement of harvesting and conversion efficiencies, an improvement in energy storage properties, as well as advanced processes for faster or simpler novel device manufacturing.

This Special Issue of *Nanomaterials* showcase state-of-the-art contributions in a broad range of subjects related to the preparation approaches and characterization techniques of (multi)functional ceramics and nanostructures in the field of energy harvesting and storage. Specifically, two research articles and four review papers are included in this Special Issue entitled “Ceramics and Nanostructures for Energy Harvesting and Storage”.

This Special Issue first presents a review paper by Bohra et al. on ZnFe_2_O_4_ as a promising, albeit not that popular, material for energy storage applications, such as photoelectrochemical fuel cells, Li-ion batteries, and supercapacitors, among others [1]. Cation disorder in inverted ZnFe_2_O_4_ nanostructures was shown to facilitate photogenerated charge separation and increase charge carrier transport in the photoanodes of fuel cells. Highly porous ZnFe_2_O_4_ nanostructures used as anodes in Li-ion batteries as well as ZnFe_2_O_4_-based heterostructures and nanocomposites with carbonaceous materials used in supercapacitor electrodes were demonstrated to be able to boost their cycle stability among other properties [1].

The next two review papers are specifically dedicated to composites of carbonaceous materials, such as graphene and reduced graphene oxide (rGO) with carbon nanotubes (CNT) [2] or polyaniline (PANI) [3], for supercapacitor electrode applications. An amount of around 10 wt.% of CNT was shown to be generally sufficient to obtain the maximum value of the specific capacitance in the case of a freestanding or substrate-supported electrode based on rGO-CNT. The addition of faradaic materials, thus forming hybrid energy storage devices, was found to result in an increase in the capacitance as well as in the highest energy and power density values [2]. At the same time, it was shown that the cycling stability of PANI was significantly increased with the addition of graphene, and the specific capacitance of graphene-related materials grew in composites with PANI, making rGO-PANI composites promising for flexible supercapacitor electrode applications [3].

The fourth review paper is related to flexible energy-harvesting applications based on materials such as BaTiO_3_ for piezoelectric nanogenerators [4]. Although at reduced output signal, flexibility and enhanced mechanical stability were achieved via the combination of BaTiO_3_ with polymers. The optimal BaTiO_3_ concentration was found to be at around 20 wt.%, while the highest value of generated power was reported for thick composite films with BT nanoparticles fabricated in the vertical orientation [4].

One of the research articles presented in this Special Issue is a study by Serrazina et al. on another lead-free piezoelectric material, K_0_._5_Na_0_._5_NbO_3_, focused on alternative sintering of its ceramics [5]. In particular, atmosphere-assisted Flash sintering was shown to be a promising technique for preparation of piezoelectric ceramics at reduced temperatures and, hence, a significantly lower thermal budget. While the low partial pressure of oxygen (reducing atmospheres) allowed a dramatic decrease in the operating temperature (T < 320 °C), an appreciable densification was obtained by using a humidified argon atmosphere and powders of nanometric particle size [5].

The Special Issue is completed with a research article by Kotarba et al. on anatase TiO_2_ nanotubes, which were obtained via anodization followed by annealing, as a material for potential solar cells and photocatalytic water-splitting applications [6]. The photoelectrochemical measurements revealed that further electrochemically reduced TiO_x_ nanotubes generated lower photocurrents during exposure to simulated sunlight compared to non-reduced TiO_2_ nanotubes, but their higher recombination time constant indicated a lower rate of electron–hole recombination under the experimental conditions [6].

In summary, this Special Issue of *Nanomaterials*, entitled “Ceramics and Nanostructures for Energy Harvesting and Storage”, compiles a series of original research articles and review papers that provide new insight into the preparation of oxide-based and hybrid nanomaterials and their wealth of applications for capacitors, supercapacitors, batteries, photoelectrochemical fuel cells, and piezoelectric energy-harvesting devices. We are confident that this Special Issue will provide readers with an overall view of some of the latest prospects in this fast-evolving and cross-disciplinary field.
